# Ability of near-infrared spectroscopy and chemometrics to predict the age of mosquitoes reared under different conditions

**DOI:** 10.1186/s13071-020-04031-3

**Published:** 2020-03-30

**Authors:** Oselyne T. W. Ong, Elise A. Kho, Pedro M. Esperança, Chris Freebairn, Floyd E. Dowell, Gregor J. Devine, Thomas S. Churcher

**Affiliations:** 1 Mosquito Control Laboratory, QIMR Berghofer Medical Research Institute, Brisbane, Queensland Australia; 2 Queensland Alliance for Agriculture and Food Innovation, The University of Queensland, St. Lucia, Queensland Australia; 3 Department of Infectious Disease Epidemiology, MRC Centre for Global Infectious Disease Analysis, Imperial College London, London, UK; 4 Private Contracting Entomologist, Hammond Island, Queensland Australia; 5 USDA, Centre for Grain and Animal Health Research, 1515 College Avenue, Manhattan, KS 66502 USA

**Keywords:** Asian tiger mosquito, Age, Spectroscopy, Chemometrics, Near-infrared

## Abstract

**Background:**

Practical, field-ready age-grading tools for mosquito vectors of disease are urgently needed because of the impact that daily survival has on vectorial capacity. Previous studies have shown that near-infrared spectroscopy (NIRS), in combination with chemometrics and predictive modeling, can forecast the age of laboratory-reared mosquitoes with moderate to high accuracy. It remains unclear whether the technique has utility for identifying shifts in the age structure of wild-caught mosquitoes. Here we investigate whether models derived from the laboratory strain of mosquitoes can be used to predict the age of mosquitoes grown from pupae collected in the field.

**Methods:**

NIRS data from adult female *Aedes albopictus* mosquitoes reared in the laboratory (2, 5, 8, 12 and 15 days-old) were analysed against spectra from mosquitoes emerging from wild-caught pupae (1, 7 and 14 days-old). Different partial least squares (PLS) regression methods trained on spectra from laboratory mosquitoes were evaluated on their ability to predict the age of mosquitoes from more natural environments.

**Results:**

Models trained on spectra from laboratory-reared material were able to predict the age of other laboratory-reared mosquitoes with moderate accuracy and successfully differentiated all day 2 and 15 mosquitoes. Models derived with laboratory mosquitoes could not differentiate between field-derived age groups, with age predictions relatively indistinguishable for day 1–14. Pre-processing of spectral data and improving the PLS regression framework to avoid overfitting can increase accuracy, but predictions of mosquitoes reared in different environments remained poor. Principal components analysis confirms substantial spectral variations between laboratory and field-derived mosquitoes despite both originating from the same island population.

**Conclusions:**

Models trained on laboratory mosquitoes were able to predict ages of laboratory mosquitoes with good sensitivity and specificity though they were unable to predict age of field-derived mosquitoes. This study suggests that laboratory-reared mosquitoes do not capture enough environmental variation to accurately predict the age of the same species reared under different conditions. Further research is needed to explore alternative pre-processing methods and machine learning techniques, and to understand factors that affect absorbance in mosquitoes before field application using NIRS.
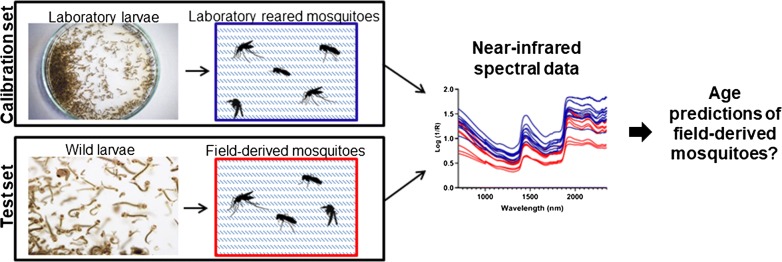

## Background

Quantifying the average age of a mosquito population would provide cost-effective and compelling entomological evidence for the potential epidemiological impacts of vector control. The mosquito death rate is the most important determinant of vectorial capacity [1] but measuring the age of wild-caught mosquitoes remains impractical and unreliable despite its epidemiological importance. The use of near infrared spectroscopy and chemometrics may offer a solution [2–6], but its ability to be used in the field remains untested.

Only older mosquitoes are able to transmit disease. This is because pathogens require time to replicate and disseminate in the mosquito after ingestion of an infected blood meal. This extrinsic incubation period (EIP) commonly takes 9–14 days for dengue and Zika [7, 8]. Age-grading methodologies are also required for determining the impact of any vector control intervention that might skew the age structure of a population (i.e. insecticide treated materials and indoor residual sprays will reduce the average age of mosquitoes). Previous methods used to age *Aedes* sp. include transcriptional profiles, morphological differences and cuticular hydrocarbon (CHC) analysis (see [9] for a review); however, these methods are often laborious, destructive, expensive and inaccurate. The work on CHCs in particular [10] gives credence to the idea that the composition of mosquito exoskeletons changes with age and that near-infrared spectroscopy (NIRS) might be used to measure the differing absorbances of those surfaces in relation to the organic compounds that they contain [10, 11]. NIRS provides information on the changing biochemical information on the surface of mosquitoes through detecting changes in C-H, N-H and O-H functional groups in mosquitoes as they age [12]. The use of NIRS to age-grade mosquitoes requires no sample preparation and is fast and accurate in distinguishing young and old mosquitoes in laboratory-derived samples. In that context, the NIRS method has been used to age grade *Anopheles* spp. [2, 5], *Ae. aegypti* [12, 13] and *Ae. albopictus* [6]. Most models to date have been laboratory-derived and typically their accuracy has been tested against mosquitoes from the same origin. The models are therefore likely to be overly optimistic. Milali et al. [14] examined spectra collected from laboratory and wild-caught *Anopheles* mosquitoes and found no significant difference between them. However, the age of the field-collected material appears to be unknown and so the capacity of those similar spectra to reflect age-related differences was not tested. At least for *Anopheles*, other studies suggest that calibrations generated with one population of mosquitoes are not applicable to combined datasets derived from NIRS studies conducted in different laboratories, on different populations or using different machines [3]. Similarly, models built using laboratory-reared mosquitoes had low predictive power in relation to the age of Anopheline adults derived from wild-caught larvae [15].

Machine learning methods are required to convert spectral data into predictive models for mosquito age. This has historically been performed using Partial Least Squares (PLS) regression and the software GRAMS IQ (Thermo Fisher Scientific, MA, USA). It has been postulated that prediction accuracy might be improved using more complex analytical and model-building techniques [15, 16] and that pre-processing data cleaning might also improve performance [3].

In the present study, we used a laboratory-reared colony of Aedine mosquitoes to attempt to predict the age of mosquitoes collected as pupae in the field and reared to known age in cages held at ambient field conditions. To our knowledge, this is the first attempt to use laboratory reared *Aedes* mosquitoes to develop predictive models of age for mosquitoes derived from field-collected material under ambient environmental conditions. We also examined whether the accuracy of our calibration and prediction models could be improved using different pre-processing and analytical techniques.

The Asian tiger mosquito (*Aedes albopictus*), which is the subject of this study, originates from Southeast Asia, but now has a global distribution facilitated by the international movement of passengers and cargo and its ability to adapt quickly to new environments [17]. *Aedes albopictus* is an important vector of dengue [18, 19] and chikungunya [20, 21]. Field female *Ae. albopictus* are on average thought to live for approximately 8 days [22] although mark-release-recapture studies suggest individual mosquitoes may live up to 17 days [23].

## Methods

### Mosquito collection and rearing

#### Laboratory-reared mosquitoe**s**

*Aedes albopictus* eggs were collected from Hammond Island, Torres Strait, Australia, in June 2016 and used to derive a stable laboratory-maintained colony at the quarantine facility in Queensland Medical Research Institute (QIMR) Berghofer. The species was first noted on the Torres Strait islands of Australia in 2005 and has since facilitated some minor dengue outbreaks in that region [24]. Larvae hatched from that colony were reared in trays (35 × 15 cm) of de-chlorinated water kept at 27 °C and 70% humidity. Larvae were fed with ground fish food *ad libitum* (Tetramin fish food flakes; Blacksburg, VA) and pupae were removed to round containers (9 cm diameter, 130 ml water). Emerging females were transferred daily to cages and provided with 10% sucrose *ad libitum* and maintained at 27 °C.

Adults used for NIRS analysis were killed 2, 5, 8, 12 and 15 days post-emergence. Individuals of the same age from two different generations were pooled to include possible variations in laboratory rearing conditions. Mosquitoes were anaesthetised with CO_2_ and placed in individual 1-ml tubes containing RNA*later*^®^ (Ambion, TX, USA), a standard protocol for NIRS characterization [25]. Tween-20 (0.1% v/v) was added to the RNA*later*^®^ to reduce surface tension and allow RNA*later*^®^ to fully penetrate the mosquito. Sample tubes were maintained at room temperature for 24 h. The mosquitoes were then preserved at − 20 °C until spectral collection (< 14 days later). The total number of mosquitoes collected is listed in Table 1.

**Table 1:** Predictive power of models derived from different *Ae. albopictus* populations

Actual age (days)	No. scanned	Standard PLS		Resampling PLS	
		Age in days	Classed as old (%)	Age in days	Classed as old (%)
Using laboratory-derived models to predict the age of laboratory-reared mosquitoes					
Number of components		8		10	
2	41	3.58 (0.28)	0	2.13 (0.22)	4
5	42	7.62 (0.30)	36	7.27 (0.23)	9
8	42	8.35 (0.27)	60	8.07 (0.21)	57
12	42	8.47 (0.33)	57	9.84 (0.22)	58
15	44	14.0 (0.33)	100	14.7 (0.26)	68
Overall accuracy		RMSD = 2.38	–	RMSD = 2.89	AUC = 0.88
Using field-derived models to predict the age of field-derived reared mosquitoes					
Number of components		8		10	
1	50	4.58 (0.32)	6	3.31 (0.28)	0
7	50	7.71 (0.44)	41	7.28 (0.36)	0
14	100	11.7 (0.34)	90	12.7 (0.26)	100
Overall accuracy		RMSD = 3.41	–	RMSD = 2.82	AUC = 0.97
Using laboratory-derived models to predict the age of field-derived reared mosquitoes					
Number of components		8		10	
1	50	6.50 (0.20)	12	8.23 (0.18)	36
7	50	6.92 (0.20)	18	9.40 (0.27)	74
14	100	7.00 (0.15)	24	9.18 (0.22)	75
Overall accuracy		RMSD = 5.84	–	RMSD = 5.42	AUC = 0.60

*Notes*: The true age of mosquito groups is shown on the left while the mean predicted age (and variability, given as standard error of the mean, SEM) is shown on the right using standard Partial Least Squares (PLS) regression or a resampling PLS framework. Mosquitoes are classified as young (< 8 days) or old (≥ 8 days). First line of each section of the table shows the number of components used in the different models. Accuracy of age estimates is shown by the average difference between the true and predicted age measured in days (root-mean-square deviation, RMSD), with lowest values indicating a more accurate model. The ability to classifying mosquitoes as young or old is given by the area under the curve (AUC), with higher values indicating greater accuracy

#### “Field-derived” mosquitoes

The term “field-derived” is used to describe mosquitoes with an origin that is more representative of the field than of the laboratory. They were collected as pupae from a natural habitat (a productive rainwater tank) on Hammond Island, Torres Strait, during March 2018. This site is also the origin of the material used to derive the 2016 laboratory colony (see above). Pupae emerged in standard rearing cages (60 × 60 × 60 cm, Bugdorm, Megaview, Taiwan) maintained outdoors under ambient conditions. Adults were aspirated from the cages when they were 1, 7 or 14 days-old, immobilized by cold (4 °C) and placed in RNA*later*^®^ with 0.1% (v/v) of Tween-20 at − 20 °C until ready for shipping to QIMR Berghofer.

### Mosquito scanning using near-infrared spectroscopy

Preserved, frozen mosquitoes were defrosted at room temperature and excess RNA*later*^®^ removed by placing specimens on paper towelling. A Spectralon plate was used for spectral background collection. Individual mosquitoes were placed on the Spectralon plate laterally, and the head and thorax were scanned using the LabSpec 5000 NIR spectrometer (Malvern Panalytical, Longmont, CO, USA). NIR spectra were obtained with an attached bifurcated fiber-optic probe that is approximately 2.4 mm above the Spectralon plate; scanning an area of approximately 2 mm. Spectral data was recorded in the 350–2500 nm region. Each spectrum was built using an average of 30 scans at a sampling resolution of 3 nm. Spectral data were collected using RS3 v6.4.3 (Malvern Panalytical, Longmont, CO, USA). Reflectance (*R*) is converted to absorbance (log 1/*R*) through RS3 prior to analyses.

### Data analysis

#### Estimating mosquito age in days

Analyses were performed within the wavelengths of 700 to 2350 nm to disregard background noise at the start and end of the spectra, and any colour differences in mosquitoes (detected at < 700 nm). PLS regression was used to convert spectral data into predictive models of mosquito age (in days). Previous mosquito NIRS studies have used GRAMS IQ software (Thermo fisher Scientific, MA, USA) to conduct the PLS analysis. GRAMS IQ uses a “leave-one-out” method for internal cross-validation where one sample is taken from the calibration set and the remaining samples are used to develop an equation that would predict that removed sample (therefore for each iteration the model is tested against a single data point). This process is repeated for all samples to create a predictive regression model (calibration model). The whole “leave-one-out” method is then repeated varying the number of PLS components (factors) and the best model selected [2, 13, 26] by choosing the number of components that maximises accuracy whilst trying to minimise overfitting (inclusion of too many components results in models that fit the sampled data perfectly but that fail to predict new data). This involves subjectively deciding when increasing the number of components starts to have a minimal impact on cross-validation accuracy. Here we repeat the methods of the past (leave-one-out internal cross-validation and selecting the number of components based on the correct classification rates of the calibration and prediction sets) and refer to this method as “Standard PLS”.

An alternative approach for the development of predictive models whilst reducing overfitting is to split the dataset into three for training, validation and testing [27]. Here we use 50% of the sample for training (fitting the model to samples of known age using different numbers of PLS components), 25% for validation (selecting an optimum number of components that effectively predict another subset of known samples) and 25% to the test dataset (evaluating the final model against a blinded subset of data). This process is repeated 100 times, each time randomly resampling the original dataset to generate different training, validation and testing datasets so that no model is validated or tested against data used in its fitting. The overall accuracy of this set of models is then reported as the mean accuracy (as measured by the root-mean-square deviation, RMSD) of the 100 different models. This averaging is necessary in order to reduce sampling noise generated by the resampling process and obtain an unbiased estimate of the error (i.e. if only a single randomisation was used accuracy could be much higher or lower by chance depending on data split). Here the number of components selected during the validation exercise (and used in all 100 models) is the lowest number of components that permits an average error (RMSD) within 0.5 days of the best fitting model. This value was arbitrarily selected to be a compromise between accuracy and generalizability (further reducing overfitting). This resampling procedure and selection of the number of components is referred to as “resampling PLS” and has been used to optimise models for predicting the presence of malaria parasites in mosquitoes [28]. Results are shown comparing the standard error of the predictions with the true age of the mosquito (RMSD). To allow a direct comparison with Standard PLS, RMSD estimates for Resampling PLS were calculated on estimates of individual mosquito age calculated from the mean of the 100 randomisations using the training/validation/test dataset. The Resampling PLS method was written for these analyses in R [29] and available from https://github.com/pmesperanca/mlevcm.

Mathematical pre-treatment of spectra may reduce noise and increase the ability of NIRS to differentiate between mosquitoes with different characteristics. To investigate whether the accuracy of the standard PLS models could be improved by pre-processing techniques we examined standard normal variate (SNV), mean normalizing, and detrend-SNV methods to minimize spectral distortion due to scattering. We used second derivative Savitzky-Golay (SG) filtering to remove baseline noise [30, 31].

#### Classifying mosquitoes as young and old

Previous NIRS studies have estimated mosquito age in days as a continuous variable and then classified mosquitoes according to whether this age estimate is above or below a pre-defined threshold (i.e. > or < X days-old; [4–6]). Here we use a binomial logistic regression framework to classify mosquitoes as young or old using the same resampling PLS framework outlined above [27]. An 8-day threshold is used to differentiate between young and old mosquitoes as it was the median age of mosquitoes collected thus allowing the calibration dataset to be evenly balanced between outcomes. Misclassification rates (the proportion of test observations incorrectly classified) were used to estimate the optimal boundary threshold (the value of the linear predictor differentiating between age classes), with sensitivity, specificity and accuracy determined using equations by Milali et al. [32]. Overall accuracy for resampling PLS is assessed by comparing the area under the receiver operating characteristic (ROC) curve (AUC). This is a graphical tool commonly used to illustrate the diagnostic accuracy of binary classification systems, with an AUC of 0.5 signifying the ability of NIRS to classify old and young mosquitoes is no better than chance whilst a value of 1 indicates perfect accuracy. The model with the minimum number of components that is within 0.01 of the model with the highest AUC is selected. Estimates of whether a mosquito is classified as young or old are made by averaging prediction of the linear predictor from 100 randomisations and comparing that to the average cut-off (in the linear predictor space) for all mosquitoes to enable a fair assessment of the quality of the model in a real-life setting [27].

#### Analysis of spectra

Potential outliers in the data were identified and removed using Hotelling T^2^ statistics, where samples positioned outside of a 95% confidence interval ellipse and consisted extreme differences in spectra are considered outliers. Outliers were not used in this analysis because they are considered data points that are not representative of the age-grading spectral information used for the development of a principal component analysis (PCA) model. Ten laboratory samples and nine field-derived samples were removed as outliers. PCA was then used to identify spectral differences and clustering within the datasets. Loading plots generated from PCA were analysed to identify key absorbance peaks that may correspond to the age grading of mosquitoes. PCA analysis was conducted in Unscrambler X (v. 10.5.1).

## Results

### Determining mosquito age in days

**Fig. 1:**
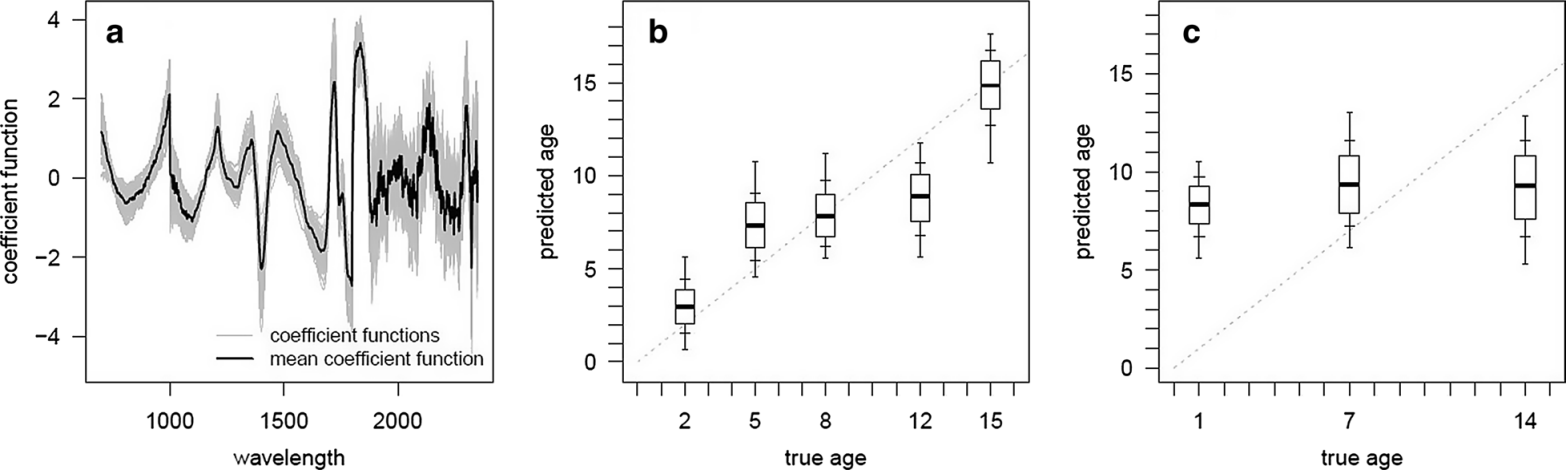
The ability of NIRS to predict the age of *Ae. albopictus* mosquitoes in days. **a** The best-fit regression coefficient function for the resampling PLS model trained on laboratory-reared mosquitoes showing the most informative regions of the spectrum. Grey lines show best-fit model for each of the 100 dataset randomisations whilst black line indicates the average. **b** Ability of the model to predict age of laboratory-reared mosquitoes. Boxplot thick horizontal black line shows the median/50th-percentile whilst the box edges, inner and outer whiskers show 25th/75th, 15th/85th and 5th/95th percentiles, respectively. Grey dashed line shows model with 100% accuracy. **c** Ability of the model trained on laboratory mosquitoes to predict the age of field-derived mosquitoes. Results can be compared to the simple PLS method presented in Additional file 1: Figure S1

NIRS can determine the calendar age of laboratory-reared *Ae. albopictus* mosquitoes with moderate accuracy but our laboratory model fails to predict the age of the same mosquito species with the same geographical origin reared *in situ*. The exact predictive accuracy depends on the method of analysis. The best fit calibration model using laboratory data are shown in Fig. 1 generated with the resampling PLS (Fig. 1a–c) and standard PLS (Additional file 1: Figure S1a–c). Both methods generate regression coefficients with peaks at similar wavelengths (Fig. 1a, Additional file 1: Figure S1a) which are broadly the same as those observed previously [12] although they differ in amplitude, and had wavelengths correspond to CH absorption overtones (1120–1225 and 1350–1450 nm). The resampling PLS framework gave an average difference between the true age and the predicted age of individual mosquitoes of 2.89 days, which was not as good as 2.38 days for the standard method (Table 1). Average estimates for the different age classes were more accurate in every age group using the resampling method, producing average age predictions that are closer to their actual age of mosquitoes compared to the standard method (Table 1).

Neither PLS model derived from laboratory-reared mosquitoes was able to predict the age of field-derived mosquitoes (Fig. 1c, Additional file 1: Figure S1c). The standard PLS method had an average error of 5.84 days whilst the resampling method gave an error of 5.42 days. Mean predicted age across the three different field-derived age groups was broadly the same, and age groups were indistinguishable from one another (Table 1). The inadequacy of using laboratory-reared mosquitoes to predict the age of field-derived mosquitoes is not driven by a lack of signal from the field-derived mosquitoes as training the model on the field-derived mosquitoes alone and then using that to predict the age of a subset of these mosquitoes (internal cross-validation) generated moderately accurate results (average error of 3.41 days and 2.82 days for standard and resampling methods, respectively, see Table 1).

Preprocessing spectra before standard PLS substantially improved the accuracy of the calibration model for the laboratory-derived mosquitoes. The most successful method was Detrend-SNV, which reduced the average error in the calibration (laboratory-derived) dataset to 2.09 days (broadly in line with the accuracy of the resampling PLS framework). However, the accuracy of that model in predicting the age of field-derived mosquitoes remained poor (average error of 4.9 days, see Additional file 2: Table S1).

### Binary classification (young or old)

The ability of NIRS to differentiate between young and old mosquitoes varied substantially according to the method of analysis (Table 1). Most previous work has classified mosquitoes as young or old by estimating the age in days and then using this to classify mosquitoes as young or old. The standard PLS model misclassified 23.8% mosquitoes reared in the laboratory with a sensitivity of 0.73, specificity of 0.82 and accuracy of 0.76, but correctly classified very young and very old laboratory-reared mosquitoes with high accuracy (100% of 2 day-old and 15 day-old mosquitoes; Table 1). However, standard PLS models derived from laboratory-reared mosquitoes failed to predict the age of field-derived mosquitoes, with a sensitivity of 0.85, specificity of 0.24 and accuracy of 0.55.

**Fig. 2:**
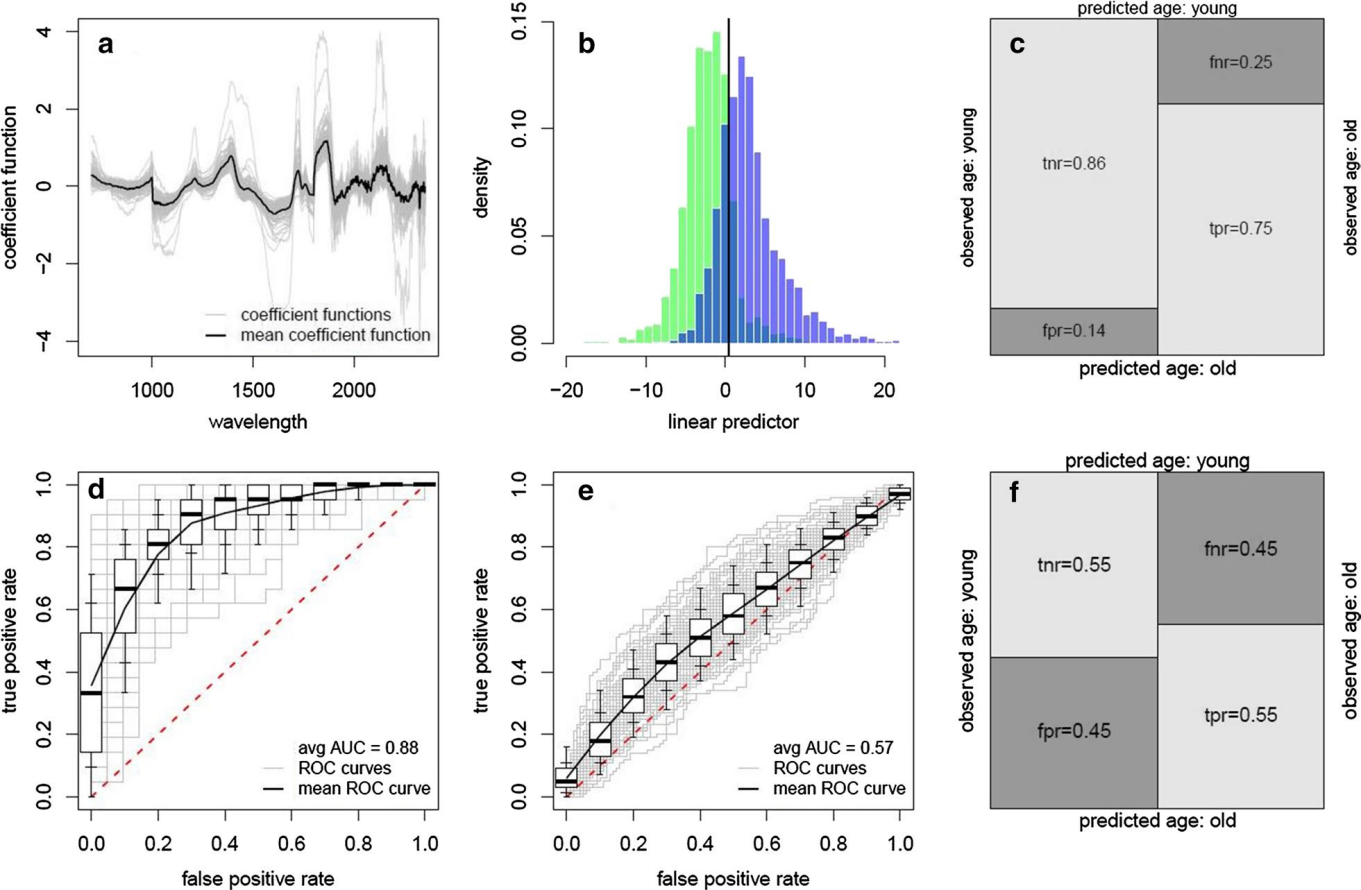
The ability of NIRS to classify *Ae. albopictus* mosquitoes as being young or old. **a** The best-fit regression coefficient function for a model trained on laboratory-reared mosquitoes showing the most informative regions of the spectrum. Grey lines show best-fit model for each of the 100 dataset randomisations whilst black line indicates average. **b** Ability of the model to predict age classification of laboratory-reared mosquitoes. Histogram of the estimated linear predictor for the test observations colour-coded by the true class (green, true young mosquitoes; blue, true old mosquitoes). Vertical black line indicates optimum threshold for classifying mosquitoes as old or young (“left” predicted to be young, “right” predicted to be old). The shaded area where two distributions overlap corresponds to misclassified test observations, false negatives to the left and false positives to the right of the optimal classification threshold. **c** The corresponding confusion matrix for the best model trained and predicting laboratory-reared mosquitoes showing the different error rates: tnr, true negative rate; fnr, false negative rate (specificity); fpr, false positive rate; and tpr, true positive rate (sensitivity). **d** The receiver operating characteristic (ROC) curve for the best-fit model predicting laboratory-reared mosquitoes showing the false positive and true positive rates achievable for different classification probability thresholds (shifting the black vertical line (**b**) left or right) whilst the overall performance is given by the area under the ROC curve (AUC). The pink dashed line denotes a model with no predictive ability (a random chance of correct prediction) whilst a perfect model with 100% sensitivity and specificity would be in the top left corner (coordinates 0, 1). The solid line shows the average ROC curve; boxplots show the variability for 100 randomisations of the training, validation and testing datasets (box edges, inner and outer whiskers show 25th/75th, 15th/85th and 5th/95th percentiles, respectively; black line inside the box showing the median/50th-percentile). **e** The ROC curve showing the ability of the model trained on laboratory rerared mosquitoes to predict the age classification of mosquitoes reared in the field-derived environment. **f** The corresponding confusion matrix of the best model

Training the model to directly classify young or old mosquitoes substantially improves the accuracy of results. The resampling PLS classification model selects different regions of the spectrum (Fig. 2a) compared to the continuous age model (Fig. 1a), though some regions were informative to both models. Overall, the ability of resampling PLS models to predict the age of laboratory mosquitoes was high (Fig. 2b–d; AUC = 0.88) with good sensitivity (0.75) and specificity (0.86). However, the model trained on laboratory mosquitoes was still unable to predict the age class of field-derived mosquitoes with a sensitivity of 0.55, specificity of 0.55 and a low overall accuracy (AUC = 0.60).

There remains a strong age-related signal from field-derived mosquitoes even if they cannot be predicted by laboratory samples. Resampling PLS models derived from field-derived samples accurately differentiated 100% of day 1, 7 and 14 mosquitoes (AUC = 0.97; Table 1). The standard PLS framework could only classify day 1 and day 14 mosquitoes with any accuracy.

### Spectra investigation

**Fig. 3:**
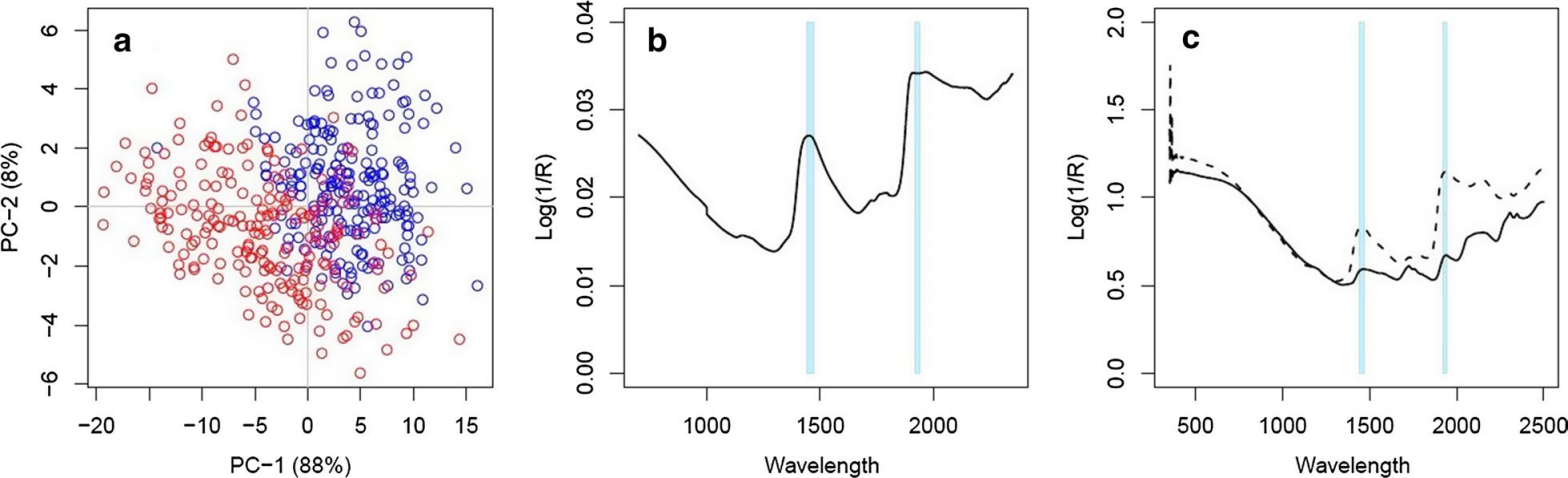
Differences between laboratory and field-derived spectra. **a** Principal components analysis showing the difference between scores calculated for PC-1 (which explains 88% of the variation) and PC-2 (8% of the variation) for laboratory (blue; square) and field-derived (red; circle) mosquitoes. **b** Loading plot from PCA showing that water bands at 1450 nm and 1930 nm accounted for 88% of the total variance observed. R denotes reflectance **c** difference between undried (dashed line) and dried (solid line) mosquito spectra. In **b** and **c** blue horizontal lines indicate peaks associated with water

Results from an analysis of spectral data identified four principal components that explained 88%, 8%, 2% and 1% of the variance observed. A scatter plot illustrates spectral differences between field-derived and laboratory mosquitoes (Additional file 3: Figure S2). The clustering of field-derived mosquitoes towards PC-1 could reflect higher water content in these samples, as variances appears to result from absorbance peaks associated with water (1450 nm and 1930 nm) [33] as can be seen in Fig. 3b. Younger mosquitoes are found to have higher water content compared to older individuals [34], indicating that water may influence age grading in insects. The water signals can be detected at these peaks when comparing signals of dried mosquitoes (storage in silica for two days) to mosquitoes treated similarly to this study (Fig. 3c). The remaining 12% of the variances observed consisted of some overtones of water absorbance peaks and many weak signals that are difficult to interpret. Scatter plots of PC-2 and PC-3 showed less dramatic spectral differences of unknown cause (Additional file 3: Figure S2). Overall, there are clear differences between spectral outputs that reflect differences in water, protein and other chemical content suggesting predicting age in field mosquitoes of different provenance will be challenging.

## Discussion

NIRS measures the absorption of organic compounds within a sample using an electromagnetic spectrum in the near-infrared region. The derived spectra are complex and multivariate analytical techniques are required for their interpretation. If these outputs are to be of utility for programmatic field evaluations, the predictive power of the derived models must effectively classify the age of field-collected material of unknown provenance. This validation is clearly challenging to design and test. It requires a comparison of NIRS data derived from mosquitoes of known calendar age, with field collected mosquitoes graded using an independent proxy such as parity, or an alternative age-grading technique such as hydrocarbon analyses or transcriptional profiles [9, 35].

An initial, simpler step in that process is to show that laboratory-derived models are applicable to field-derived material. In this instance, we attempted to correlate the calendar age of mosquitoes from a laboratory colony, with the calendar age of mosquitoes derived from field-collected pupae reared to the adult stage in cages held at ambient field conditions.

We determined that PLS regression models can predict the age of other mosquitoes reared under near-identical conditions. This is consistent with other studies that have used models derived from laboratory-reared *Ae. albopictus*, *Ae aegypti* and *Anopheles gambiae* (*s.l.*) colonies to predict the age of other mosquitoes from the same colonies [6, 12, 13, 36]. In an extension of that work, our study demonstrates that models derived from laboratory mosquitoes were unable to predict the age of field-derived mosquitoes. Similar results were obtained when mosquitoes were ascribed a binary age classification (< or > 8 days). This has important implications as the utility of the technique in field programmes will rely upon an ability to use data sets from one origin, geographical area or sampling period to accurately predict the age of other samples of a different provenance.

Although our laboratory and field-derived mosquitoes originated from the same site, the former was established in 2016 and is likely to have diverged significantly in profile from those mosquitoes collected in 2018. Their respective histories of nutrition, competition and development times will have been very different, as will a host of other environmental and abiotic factors. We therefore cannot determine whether the failure of models trained on field data to predict the age of field-derived mosquitoes is due to differences in the mosquito population or the rearing conditions. Further work is needed to confirm whether models derived from laboratory-reared mosquitoes can be used to predict the age of field-derived, semi-field and field mosquitoes. The utility of NIRS will depend on whether models trained on one group of mosquitoes would be able to predict the age of mosquitoes from different times and places. PCA analysis suggest substantial variation in spectra between laboratory- and field-derived material. That seems at odds with the conclusions of a previous study, which suggests no difference between near infrared spectra collected from laboratory and field mosquitoes [14]. Spectral analyses suggest that water absorbance peaks may contribute to the variation observed here, and reflect the physiological state of the mosquito or the immediate environment. Water creates strong NIRS signals that may dominate other signatures in the cuticle [16] and may be masking important, age-related spectra; however, this cannot be confirmed unless there are additional studies performed on dried mosquitoes. All adult mosquitoes used in this study were cage-reared with *ad libitum* access to 10% sugar solution; therefore, it is unknown if water signals directly influences spectral data collected for age grading, and whether moisture content in a mosquito is a limitation for NIR mosquito studies. There were also absorption peaks indicating differences in CH absorption overtones in the best-fit regression coefficient function for standard and resampling PLS methods, which has been reported to be important in age classification of other insects [37].

The preservation of mosquitoes with the use of Tween-20, although used in very small amounts (0.1% v/v), may have the potential to remove lipids and wax from the surface of mosquitoes. This could have the potential to affect NIR signals however all mosquitoes were preserved similarly to avoid variation between samples, and results obtained from cross-validation had similar outcomes to a previous study on *Ae. albopictus* [6]. Additionally, changes in components other than epicuticular lipids have been proven to contribute significantly to changes in NIR signals in aging insects [37].

Exploring alternatives to the standard PLS regression improved internal cross-validation in some cases. The resampling method which uses 100 randomisations of the original dataset substantially reduces overfitting, especially for relatively small datasets [27]. This study used spectra from ~40 mosquitoes of highly homogenous origin to represent each age category. This is in line with previous studies [2, 6, 13, 26] but the accuracy and robustness of machine learning techniques will improve substantially as more samples are included and as more variability is captured [3]. The resampling PLS framework also enables the number of components to be automatically selected, increasing reproducibility and probably contributing to improvements in the out-of-sample accuracy [28]. Pre-processing of spectral data also appeared to improve accuracy of the standard PLS and is routinely applied to spectral data, especially on solid materials where light scattering often occurs [38]. This reduces background noise, which consists of random deviations of the spectral measurements and systematic variations within samples which are unimportant in the analysis [39]. In our study, pre-processing produced calibration models with a higher accuracy and fewer principle components for laboratory-derived calibration predicting ages of laboratory-reared mosquitoes, and field-derived calibration predicting ages of field-derived mosquitoes. There were no significant differences in accuracy of field-derived age predictions using laboratory calibrations but there seems further utility in exploring alternative pre-processing methods and machine learning techniques.

## Conclusions

There remain many challenges to the development and adoption of NIRS as an age-grading tool with a field application. The application of NIRS and chemometrics to the age classification of insects would benefit from a better understanding of the factors that affect absorbance and the challenges they pose to accurate prediction. A spectral database defined in terms of its causative physiological or biochemical drivers might allow for data analyses to be performed using only the most relevant regions, as can be seen in a mosquito mid-infrared study [16]. This method has also been used in various other NIRS studies, where there is emphasis on an individual wavelength related to the detection of a specific chemical bond [40, 41]. Efforts to define its potential and limitations are essential as we consider our priorities for research and development.

## Supplementary information

**Additional file 1: Figure S1.** The ability of NIRS to predict the age of *Ae. albopictus* mosquitoes in days. **a** The best fit regression coefficient function for the simple PLS model trained on laboratory-reared mosquitoes showing the most informative regions of the spectrum. **b** Ability of the model to predict age of laboratory-reared mosquitoes. Boxplot thick horizontal black line shows the median/50th-percentile whilst the box edges, inner and outer whiskers showing 25th/75th, 15th/85th and 5th/95th percentiles, respectively. Grey dashed line shows model with 100% accuracy. **c** Ability of the model trained on laboratory mosquitoes to predict the age of field-derived mosquitoes. Results can be compared to the resampling PLS method presented in Fig. 1.

**Additional file 2: Table S1.** Impact of pre-processing on standard PLS calibration models. Accuracy is compared using the mean average difference between the predicted and true age (root-mean-square deviation, RMSD, in days).

**Additional file 3: Figure S2.** Scatter scores plot for laboratory (blue; square) and field-derived (red; circle) mosquitoes generated from PCA models. **a** Scores calculated for PC-2 (8%) and PC-3 (2%). **b** Scores calculated for PC-3 (2%) and PC-4 (1%).

## Data Availability

Not applicable.
