# Supplementary figures and images for: Ability of near-infrared spectroscopy and chemometrics to predict the age of mosquitoes reared under different conditions
Source: Parasit Vectors. 2020 Mar 30;13:160. doi: 10.1186/s13071-020-04031-3 (PMC7106667; doi:10.1186/s13071-020-04031-3)

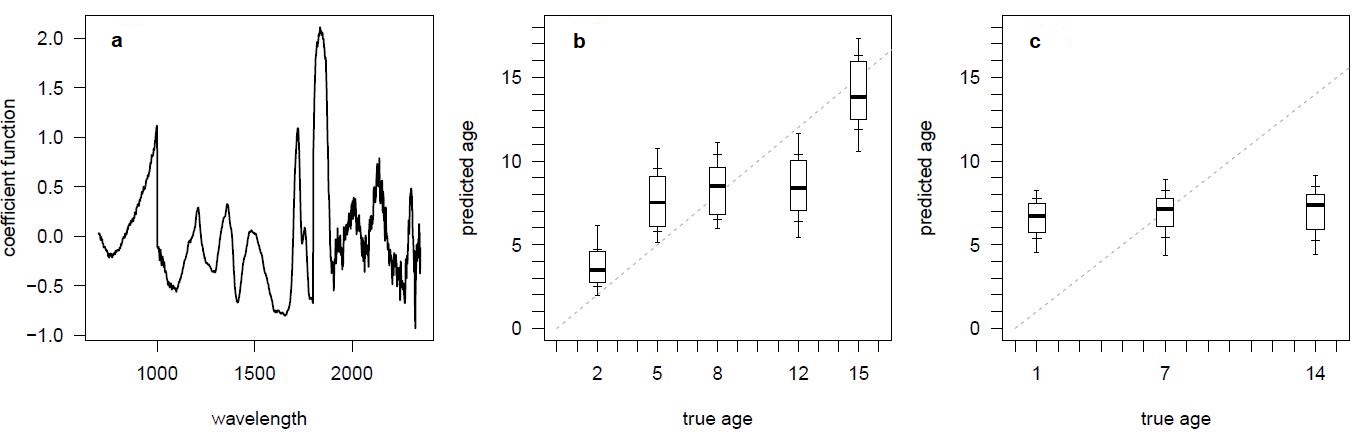

Supplement: Supplementary file 1 — Additional file 1: Figure S1. The ability of NIRS to predict the age of Ae. albopictus mosquitoes in days. a The best fit regression coefficient function for the simple PLS model trained on laboratory-reared mosquitoes showing the most informative regions of the spectrum. b Ability of the model to predict age of laboratory-reared mosquitoes. Boxplot thick horizontal black line shows the median/50th-percentile whilst the box edges, inner and outer whiskers showing 25th/75th, 15th/85th and 5th/95th percentiles, respectively. Grey dashed line shows model with 100% accuracy. c Ability of the model trained on laboratory mosquitoes to predict the age of field-derived mosquitoes. Results can be compared to the resampling PLS method presented in Fig. 1. [file 13071_2020_4031_MOESM1_ESM.jpg]

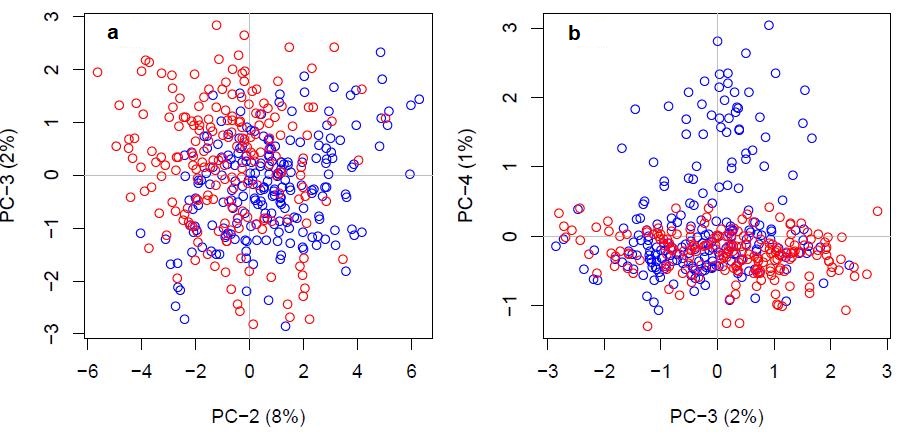

Supplement: Supplementary file 3 — Additional file 3: Figure S2. Scatter scores plot for laboratory (blue; square) and field-derived (red; circle) mosquitoes generated from PCA models. a Scores calculated for PC-2 (8%) and PC-3 (2%). b Scores calculated for PC-3 (2%) and PC-4 (1%). [file 13071_2020_4031_MOESM3_ESM.jpg]
