# Supplementary material for: Ability of near-infrared spectroscopy and chemometrics to predict the age of mosquitoes reared under different conditions
Source: Parasit Vectors. 2020 Mar 30;13:160. doi: 10.1186/s13071-020-04031-3 (PMC7106667; doi:10.1186/s13071-020-04031-3)
Supplement: Supplementary file 2 — Additional file 2: Table S1. Impact of pre-processing on standard PLS calibration models. Accuracy is compared using the mean average difference between the predicted and true age (root-mean-square deviation, RMSD, in days). [file 13071_2020_4031_MOESM2_ESM.docx]

**Additional file 2: Table S1.** Impact of pre-processing on standard PLS calibration models. Accuracy is compared using the mean average difference between the predicted and true age (root-mean-square deviation, RMSD, in days).

| **Pre-processing method** | **Laboratory mosquitoes** | | **Field-derived mosquitoes** | |
| --- | --- | --- | --- | --- |
|  | **Number of components** | **RMSD** | **Number of components** | **RMSD** |
| Mean-centred | 7 | 3.12 | 7 | 4.22 |
| Mean normalize | 6 | 3.04 | 2 | 4.18 |
| SNV | 7 | 2.83 | 2 | 4.13 |
| Detrend-SNV | 9 | 2.35 | 12 | 3.10 |
| Savitzky-Golay (2,2,28,57)* | 3 | 2.57 | 7 | 3.60 |

*Numbers within SG filtering represents the order of derivative, polynomial order, smoothing points for each sides of the plot and total smoothing points.
